# Mechanical, Transportation, and Microstructural Characteristics and Application of High-Porosity Coal Mine Solid Waste Filling Materials: A Case Study

**DOI:** 10.3390/ma18225098

**Published:** 2025-11-10

**Authors:** Qiang Sun, Hongzhen Nie, Yong Han, Rui Zhao

**Affiliations:** 1 School of Mines, China University of Mining and Technology, Xuzhou 221116, China; nhz0910@126.com (H.N.); 17616130252@163.com (Y.H.);; 2 Key Laboratory of Deep Coal Resource Mining of Ministry of Education, Xuzhou 221116, China

**Keywords:** foam filling material, coal gangue, fly ash, cell structure, abandoned roadway

## Abstract

The disposal of coal mine solid waste has always been a challenge in the coal mining production process, and the research and development of low-cost and high-performance filling materials is a prerequisite for achieving large-scale disposal of coal mine solid waste. The effects of water–cement ratio, foaming agent dilution ratio, foam agent content, foam stabilizer content, and gypsum content on the mechanical properties, transportation characteristics, and microstructure of cement foam filling materials were studied by laboratory test methods. The optimal ratio of cement foam filling material for comprehensive performance was determined. On this basis, the mechanism of influence of fly ash content, gangue content, and gangue particle size on the mechanics, transportation characteristics, and microstructure of foam filling materials was further studied. The experimental results show that at fly ash contents below 30%, gangue content is less than 30%. The particle size of gangue is less than 0.6 mm, and the expansion ratio of coal mine solid waste foam filling material is about three times, which has good mechanical properties and transportation performance. The on-site test results show that the control effect of the surrounding rock in the goaf is good, achieving safe and efficient mining of the working face.

## 1. Introduction

Coal remains China’s main energy source [1], accounting for over 60% of energy consumption in 2024 and reaching 4.76 billion tons. Experts predict that China’s coal demand will be about 3.5~4 billion tons by 2030, and the position of coal as the main energy source will not change in the short term [2]. However, as the world’s largest producer and consumer of coal, China’s coal mine solid waste problem has become a prominent contradiction restricting the green transformation of energy. The cumulative stockpile of coal mine solid waste (such as coal gangue and fly ash) has reached tens of billions of tons. In 2023, coal gangue and fly ash productions in China’s coal industry were 808 and 899 million tons, accounting for more than half of the industrial solid waste production. The coal mine solid waste stockpile currently occupies about 15,000 hectares of land [3]. The open-air stacking of coal gangue is highly prone to spontaneous combustion, releasing toxic and harmful gases. When exposed to wind, sun, and rain, the toxic heavy metals in solid waste 80 can also seep into the nearby soil through rainwater leaching and wind force, causing serious pollution to the mining area and its surrounding areas [4]. The large-scale disposal and resource utilization of coal mine solid wastes are still challenging for China [5]. In 2023, the comprehensive utilization rate of typical bulk industrial solid wastes such as coal gangue and fly ash in China was only 53.32%.

Coal mine solid waste as a filling material is an important means to achieve green mining and solid waste resource utilization in mines. It is widely used in the control of filling rock layers [6], water resource protection [7,8], surface subsidence control [9], and other fields. Guo et al. [10] used coal-based solid waste as aggregate, gypsum and alkali activator as auxiliary materials and added a small amount of additives to conduct macro- and microscale experiments on the materials, and determined the optimal ratio of coal gangue powder, slag, and fly ash. Xu et al. [11] studied CO_2_ mineralization filling materials at room temperature and pressure to achieve CO_2_ sequestration, solid waste treatment, and overburden movement control. The materials’ uniaxial compressive strength (UCS) peaked at a fly ash ratio of 75% and exhibited good flowability. Still, the fly ash ratio was inversely proportional to the CO_2_ mineralization rate. Zhang et al. [12] conducted uniaxial compression tests and scanning electron microscopy (SEM) observations on samples with different coal gangue particle sizes and fly ash ratios. The results showed that the UCS and elastic modulus decreased as the fly ash to large-sized gangue ratio increased. Under uniaxial compression load, the failure is usually in the form of tensile failure. At the same time, samples with high proportions of fly ash and large particle size gangue are more prone to shear failure. Sun et al. [13] used high-water-content materials as bonding materials and coal gangue as aggregates to study the mechanical properties and damage evolution of coal gangue and high water–material bonding fillers under the influence of three factors: coal gangue particle size, coal gangue content, and water–cement ratio. The results showed that the material has high residual strength.

Foam filling material is lightweight, highly efficient, offers a significant cost advantage, and is green and environmentally friendly. Compared with ordinary concrete, it can save 30~50% of raw material consumption, its density is only 1/5~1/3 of ordinary concrete, and it has good mechanical properties. Recently, it has been widely used in mine filling [14]. Relevant scholars have carried out a lot of research. Zhang et al. [15] developed a lightweight coal gangue foam concrete. The effects of water–binder ratio, gangue content, fly ash content, and foam content on the UCS, dry density, water absorption, and porosity of gangue foam concrete were analyzed through single-factor and orthogonal tests. They reported that the water–binder ratio and the foam content were the two key factors controlling the mechanical properties of the specimens. The coal gangue content was a key factor affecting the porosity of the specimen: at a 30% gangue content, the porosity performance index of the specimens was optimal. Pan et al. [16] produced ultra-low-density foam concrete by introducing additives. The UCS of 28-day foam concrete ranged from 0.33 to 1.1 MPa. Liu et al. [17] verified a new high-efficiency foaming agent with a high foam expansion rate and stable foam. After 28 days of curing, the base shows high UCS, low thermal conductivity, and low water absorption. In addition, numerous scholars have conducted extensive research on cost savings, flowability, water absorption, and other aspects [18,19,20].

At present, few coal mine solid wastes, such as gangue, fly ash, and foam materials, are mixed for filling and utilization, which can not only dispose of solid wastes but also reduce the cost of traditional cementation filling materials. Based on the study of the mechanical, transportation, and microstructure characteristics of different influencing factors of cement foam filling materials, this paper focuses on the influence mechanism of fly ash content, gangue content, and gangue particle size on the performance of foam filling materials. It determines the superior ratio of coal mine solid waste foam filling materials in combination with the microstructure of material foam pores, providing support for the development and large-scale disposal of coal mine solid waste filling materials in coal mines.

## 2. Materials and Methods

### 2.1. Materials

The selection of appropriate base and auxiliary raw materials is crucial for filling materials with good mechanical characteristics. In this study, ordinary Portland cement 42.5 R (P.C 42.5), coal gangue, and fly ash were selected as the base materials, while foaming agent, gypsum, and foam stabilizing agent were used as auxiliary materials to prepare a foam filling material with a high expansion rate and good mechanical properties. The fly ash and coal gangue were acquired from Baodian Coal Mine in Jining City, Shandong Province, China. A D8-ADVANCE diffractometer (Bruker, Karlsruhe, Germany) with copper target anode material, high-flux K-alpha-1 radiation, 40 kV X-ray tube voltage, 30 mA X-ray tube current, a goniometer radius of 250 mm, and a 0.019450-step sampling interval was used in the X-ray diffraction (XRD) tests [21]. Adding α-Al_2_O_3_ to the tested sample can simultaneously obtain the diffraction peak signals of the tested component and α-Al_2_O_3_ through X-ray diffraction (XRD) technology, and then calculate the content of the tested component by comparing the signal intensities. Their chemical compositions are listed in Table 1.

As seen from Table 1, the main chemical component of Baodian coal mine fly ash was SiO_2_, and the main mineral component was quartz, followed by kaolinite. The SiO_2_ content exceeded 50%. The main chemical component of Baodian coal mine gangue was SiO_2_, with quartz as the main mineral component, followed by kaolinite and diopside.

As a regulator of the hydration process, gypsum can inhibit the dissolution rate of silicate minerals, delay the setting and hardening cycle, and optimize the microstructure and density of materials. Hard gypsum was selected for this experiment, and its chemical composition is listed in Table 2.

This experiment used an HTQ-I type composite foaming agent produced in Jinan Qicai Chemical Co., Ltd., Jinan, China. This foaming agent is a transparent, viscous liquid with a slight odor. After dissolving in water, it becomes a light, white, transparent liquid with an excellent foaming effect. The bubbles are stable, independent, and evenly distributed. The foam stabilizer used in the experiment is calcium stearate, with the following molecular formula: Ca(C_17_H_35_COO)_2_. Its chemical composition is tabulated in Table 3.

The use of foam materials in the preparation of bubbles can promote bubble formation, improve their stability, and make their size distribution more uniform.

### 2.2. Preparation Method of Foam Filling Material

**Figure 1:**
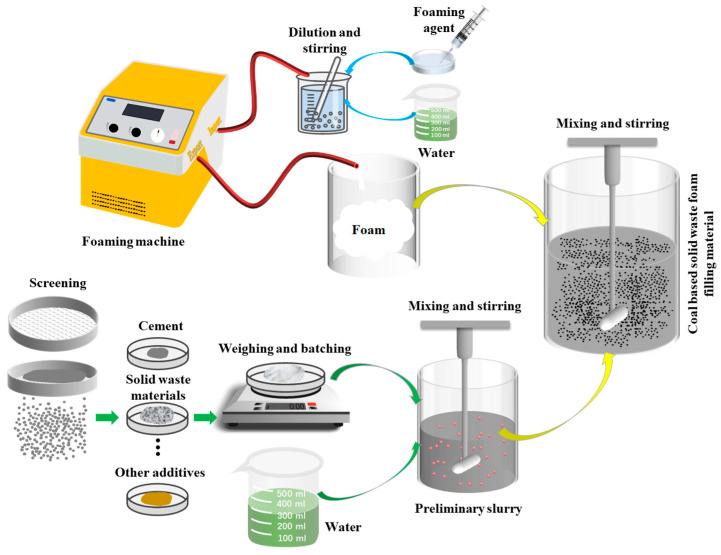
Schematic diagram of the coal mine solid waste foam filling material preparation process.

The preparation process of coal mine solid waste foam filling material includes slurry preparation and foam preparation [22]. Then the two are mixed to form a coal mine solid waste foam filling material. The main steps are as follows: Weigh the raw materials according to the designed experimental ratio, and add them to the mixing bucket with water in the set ratio, stirring evenly to form a slurry. Then, a measuring cylinder is used to weigh the corresponding amount of foaming agent and dilute it, and the diluted foaming agent is compressed through the air of the foaming machine to form foam. Finally, mix foam and slurry for 1~2 min, and the rotating speed should be 1000 rpm to make coal mine solid waste foam filling material. According to the performance test requirements, the mixed coal mine solid waste foam filling material is injected into the mold. After leaving the formed specimen at room temperature for 24 h, demold it and place it in a curing box for curing until the test age. The material preparation process is shown in Figure 1.

### 2.3. Optimal Dilution Ratio of Foaming Agent

**Figure 2:**
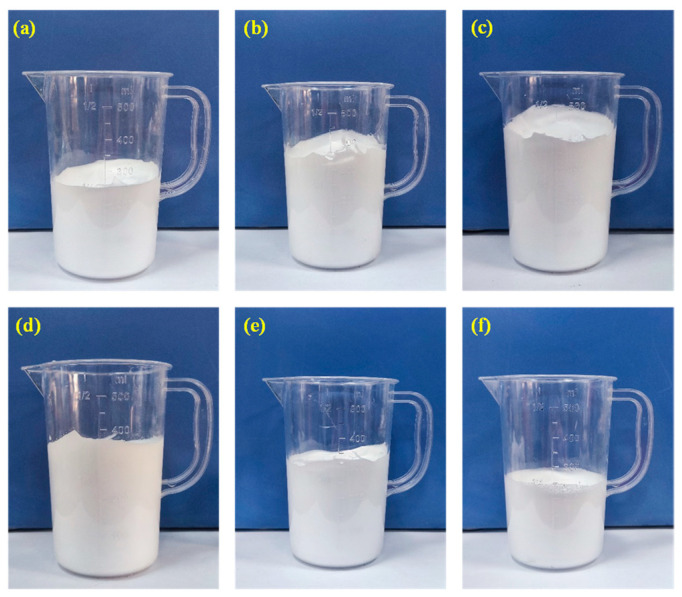
Foaming amounts of foaming agent solutions with different concentrations: (**a**) 50 times; (**b**) 40 times; (**c**) 30 times; (**d**) 20 times; (**e**) 10 times; (**f**) 5 times.

The dilution ratio of the foaming agent is crucial for the foaming effect, including foaming volume, bubble size, and uniformity [23,24]. This experiment first determines a reasonable dilution ratio of the foaming agent through comparative experiments. The main experimental method is diluting the foaming agent with different multiples and foaming through the machine. The foam produced by the corresponding concentration of foaming agent solution is introduced into the container of the same specification. The height of the foam in the container is observed, and the foaming amount is compared according to the height. The main experimental steps include diluting the foaming agent solution five times, starting the foaming machine (Quanyu Machinery, Hebei province, China), and foaming the solution. Lead the generated foam into the glass container through the hose, take photos, and observe the foaming condition of the foaming agent. Prepare foaming agent solutions of other concentrations and perform foaming. Study the foaming situation of different concentrations of foaming agents, and compare to determine the optimal dilution ratio. When the dilution ratio is 50 times, 40 times, 30 times, 20 times, 10 times, or 5 times, the foaming behavior of the foaming agent is shown in Figure 2.

The results show that the dilution ratio of the foaming agent significantly correlates with the foam’s stability and structural characteristics. When the dilution ratio is low, the interfacial active substances are not fully dispersed, resulting in a gas–liquid interface blockage, which is manifested by the limited volume of foam and high dispersion of pore size distribution. As the dilution ratio is increased to 10 times, the solution concentration approaches the optimal foaming range, and the orderly arrangement ability of surfactant molecules is enhanced, forming a foam with a thickened foam wall and significantly expanded volume. The optimal equilibrium state is reached when the dilution ratio is 20 times. At this time, the solution concentration can ensure the full diffusion of surfactant and achieve uniform pore size and dense foam structure. When the dilution ratio is more than 30 times, the surfactant concentration is insufficient, leading to the foam’s volume shrinkage and structural fragility. Especially when the dilution ratio is more than 40 times, the imbalance of interfacial tension causes many foam bursts. Overall, a dilution ratio of 20 times can balance the foaming effect and finished product quality.

### 2.4. Optimal Selection of Foam Stabilizer Dosage

**Figure 3:**
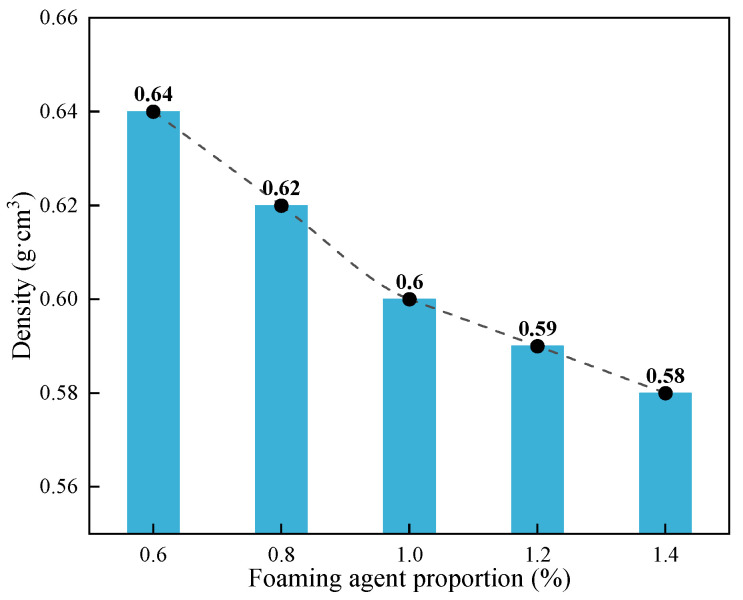
Effect of foam stabilizer proportion on the material density.

When a certain amount of foam stabilizer is added to prepare foam filling material, bubble formation, uniform size, and stability are significantly improved [25,26]. Then the expansion ratio of the material is affected. The experimental method is to add different amounts of foam stabilizing agent under a certain dilution ratio of foaming agent when preparing foam filling materials and select the optimal amount of foam stabilizing agent according to the density of the prepared specimen. The main experimental steps include weighing the raw materials according to the designed experimental ratio, adding them to the mixing tank in the set ratio with water, adding different amounts of foam stabilizers, and stirring evenly to form a slurry. The particular steps are as follows. Weigh the corresponding amount of foaming agent with a measuring cylinder and dilute it. Compress the diluted foaming agent through the air of the foaming machine to form foam. Mix foam and slurry to make foam filling material, and inject the mixed foam filling material into the mold. After placing the formed specimens at room temperature for 24 h, demold them and record the dimensions and weight of length, width, height, etc. The density was obtained based on the mass and volume of the sample. The density test results of cement-based foam materials with different contents of foam stabilizer are shown in Figure 3 and listed in Table 4.

The experimental results indicate that with the increased stabilizer dosage, the density and mass of the specimens show a decreasing trend, while the expansion ratio increases. This is because the addition of foam stabilizer optimizes the dynamic stability of foam, making the generated foam last longer and less prone to breaking under the same foaming efficiency, which will reduce the loss rate of foam when mixing and making parts, thus leading to a decrease in density and an increase in expansion ratio. When the dosage of foam stabilizer grew from 0.6% to 1%, the specimen’s density decreased significantly, and the expansion increased significantly, by about 2.2 times from 1% to 1.2%. Therefore, a foam stabilizer dosage of 1% is more suitable. When the cement slurry was not foamed and the specimen’s density was about 1.8~1.9 g·cm^−3^, the expansion ratio of the specimens in this experiment was more than three times. The dosage of stabilizer for subsequent experiments was chosen to be 1%.

### 2.5. Material Performance Testing Method

The density of foam filling materials is an important indicator that reflects the material’s pore volume and expansion performance; materials with low density and light weight are beneficial for their transportation and application. Meanwhile, UCS is a commonly used indicator to reflect the mechanical properties of materials, which is of great significance for meeting the filling requirements and achieving the filling effect. This experiment used a WAW-1000D electro-hydraulic servo universal testing machine (Changchun Xinte Testing Machine Co., Ltd., Changchun, China). The material’s slump and initial setting time directly reflect its flow effect and conveying characteristics. Therefore, this experiment mainly tests the characteristics of material density, UCS, slump, and initial setting time. Finally, electron microscopy scanning experiments studied the pore characteristics and density of different foam filling materials, revealing the characteristics of different foam filling materials and determining the optimal ratio. The microscopic characteristics of materials directly reflect their mechanical and chemical properties and play an important role in fatigue, fluidity, and processing technology.

## 3. Characterization of Cement Foam Filling Materials

### 3.1. Mechanical and Transportation Properties Test

(1) Water–cement ratios

**Figure 4:**
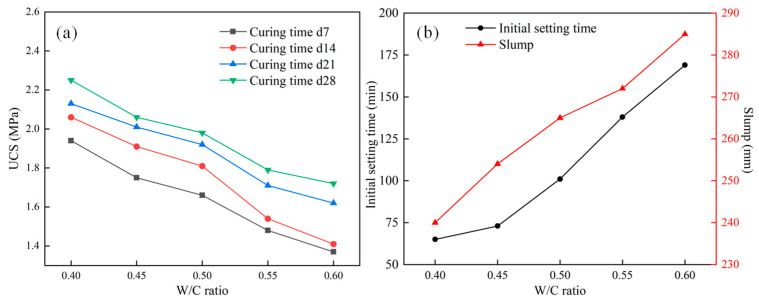
Variations in the UCS and initial setting time with different water–cement ratios: (**a**) UCS, (**b**) initial setting time.

The water–cement ratio is a key factor affecting foam filling materials’ fluidity and initial setting time [27]. A low water–cement ratio can lead to insufficient bonding of dry powder in the filling material, poor fluidity, and easy blockage in the pipeline. Although a high water–cement ratio can improve the fluidity and pumpability of the slurry, excessive moisture can prolong the setting time of the filling material. After the material hardens, the evaporation of moisture can form irregular pores in the filling body, affecting the later strength. In this section, based on the dilution ratio of 20 times for the foaming agent and the dosage of 1.0% for the stabilizer, the focus is on studying the initial setting time, slump, UCS, and other characteristics of foaming filling materials under different water–cement ratios. The test results are listed in Table 5 and depicted in Figure 4.

It can be seen from Table 5 and Figure 4 that when UCS tests were conducted on cement-based foam specimens prepared at different water–cement ratios, the UCS of the specimens showed a gradual decline trend with the increase in water-cement ratio. Although the amount of foaming agent and foam stabilizing agent in the specimens prepared at different water–cement ratios was constant, when the water–cement ratio was low, the proportion of cement was larger, and the cementation performance of the materials was stronger. At a curing period of 28 days and a 0.4 water–cement ratio, the UCS of the specimens reached the maximum value of 2.25 MPa. At water–cement ratios of 0.45, 0.5, 0.55, and 0.6, the UCS values were 2.06, 1.98, 1.79, and 1.72 MPa, respectively. The slurry’s initial setting time and fluidity gradually increased with the water–cement ratio. At water–cement ratios below 0.5, the initial setting time and slump increased slowly and then accelerated. However, with the water–cement ratio increased, the cement-based foam material slurry gradually changed to a paste, and the cementation performance gradually weakened. Generally, at a 0.5 water–cement ratio, the slurry had the best fluidity and cementation. Therefore, a moderate water–cement ratio can effectively ensure that cement has good flowability after sufficient hydration while considering the required material bearing characteristics.

(2) Foaming agent proportion

**Figure 5:**
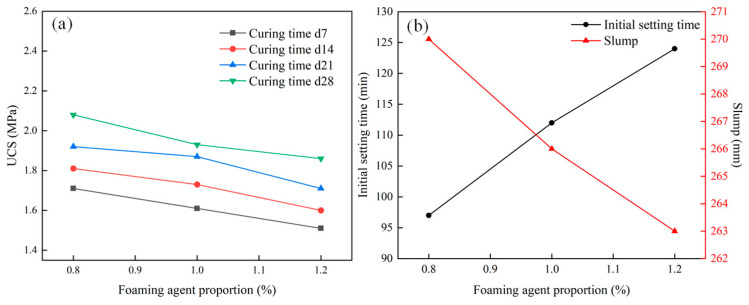
Variations in the UCS and initial setting time with foaming agent proportion: (**a**) UCS, (**b**) initial setting time.

The amount of foaming agent directly determines the foaming degree of the material, significantly impacting the density, UCS, and flow performance of foam filling materials. Based on determining the water–cement ratio of 0.5, this experiment added different amounts of foaming agent, compressed the air of the foaming machine to make it produce foam and expand, and then mixed with the cement slurry to make cement foam filling material. The material ratio and material characteristic test results are shown in Table 6 and Figure 5.

According to Table 6 and Figure 5, the following can be seen:

(a) When the water–cement ratio is constant, the performance test results of cement foam filling materials prepared with different foaming agent contents show that the initial setting time is positively related to the foaming agent content, while the slump is negatively related. The main reason is that with the increase in foaming agent content and surfactant concentration, the cell volume increases rapidly, while the fluidity of the foam is poor. With the increase in foam, the fluidity of the slurry will gradually decrease.
(b) From the perspective of physical and mechanical properties, when the dosage of foaming agent is too high, the expansion ratio also increases, increasing the porosity of the cement slurry in the free state, relaxation of the structure, and a decrease in strength. As the curing period increases, the UCS of the specimen gradually increases. With the increase in foaming agent concentration, the UCS of the specimen continuously decreases. When the foaming agent dosage is 0.8% and the curing period is 28 days, the UCS reaches 2.08 MPa. When the foaming agent dosage is 1% and the curing period is 28 days, the UCS reaches 1.93 MPa. At a foaming agent dosage of 1.2% and a curing period of 28 days, the UCS reaches 1.86 MPa. Macroscopically, it manifests as an increase in the specimen’s size and quantity of holes.
(3) Gypsum proportion

**Figure 6:**
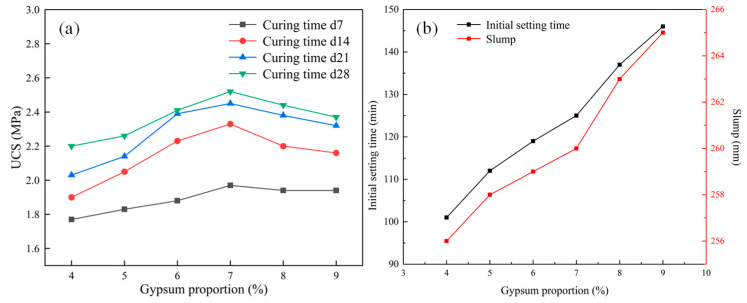
Variations in the UCS and initial setting time (**b**) with gypsum proportion: (**a**) UCS, (**b**) initial setting time.

When a certain amount of gypsum is added to ordinary Portland cement, it can not only adjust the hydration rate of composite cement and affect the setting time of the slurry but also enhance the later strength of the filling material, significantly affecting the flow and mechanical properties of the material. The material ratio and material characteristic test results are presented in Table 7 and Figure 6.

According to Table 7 and Figure 6, the following can be seen:

(a) With the increase in gypsum content, the UCS of the samples first increases and then decreases. At a curing time of 28 days and a gypsum content of 7%, the UCS of each group of samples reaches the maximum value of 2.52 MPa. When the gypsum content increases from 4% to 7%, the UCS increases by 18%, 5%, and 3% at 14, 21, and 28 days, respectively. When the gypsum content exceeds 7%, the sample’s UCS drops with the increased gypsum content.
(b) The main function of gypsum in Portland cement is to inhibit the rapid hydration reaction of calcium aluminate in cement, adjust the setting time, and an appropriate amount of gypsum can also improve the long-term strength of cement. The material’s initial setting time and slump increase with increased gypsum content. This is because gypsum can improve the workability of cement slurry, increase the fluidity of slurry, and also react with calcium aluminate in cement to generate calcium aluminate sulfate, which can inhibit the rapid hydration reaction of calcium aluminate and prolong the setting time of cement.

In conclusion, based on the test results of transportation performance and mechanical properties of cement foam filling materials, it is determined that the superior ratio of the comprehensive performance of cement foam filling materials is that the dilution concentration of foaming agent is 20 times, the content of foam stabilizer is 1%, the water–cement ratio is 0.5, the content of foaming agent is 1%, and the content of gypsum is 7%.

### 3.2. Microstructural Examinations

**Figure 7:**
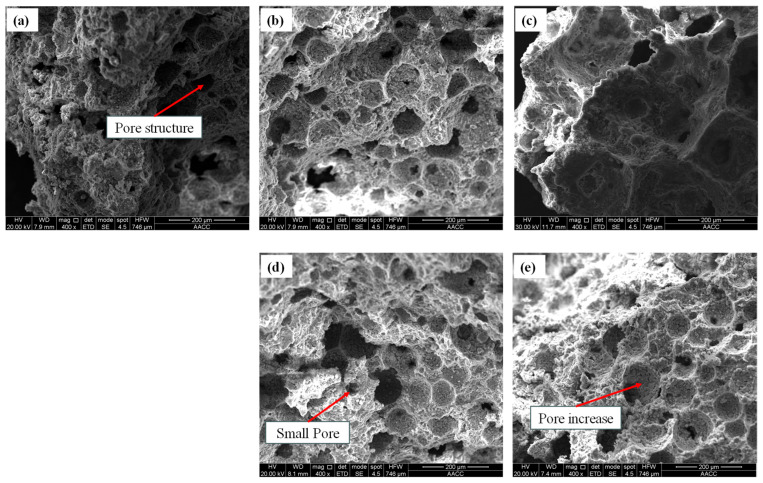
SEM test results of cementitious foam filling materials with different water–cement ratios: (**a**) 0.4, (**b**) 0.45, (**c**) 0.5, (**d**) 0.55, (**e**) 0.6.

**Figure 8:**
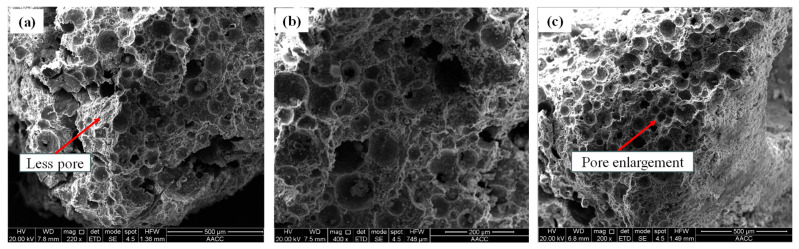
SEM test results of cementitious foam filling materials with different foaming agent dosages: (**a**) 0.8%, (**b**) 1%, (**c**) 1.2%.

**Figure 9:**
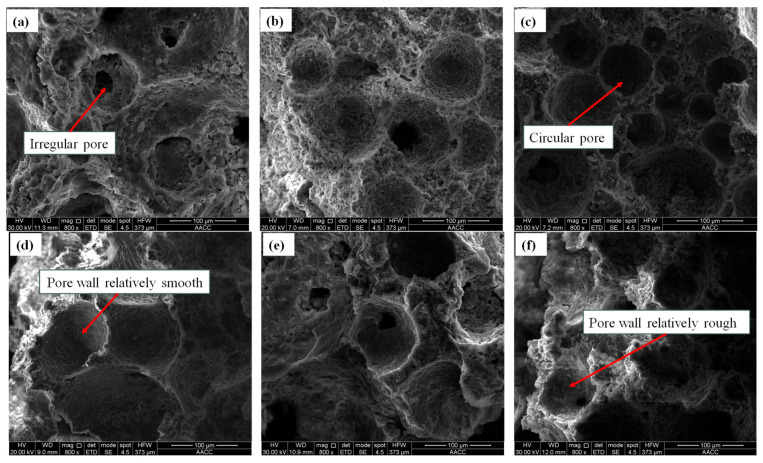
SEM test results of cementitious foam filling materials with different gypsum dosages: (**a**) 4%, (**b**) 5%, (**c**) 6%, (**d**) 7%, (**e**) 8%, (**f**) 9%.

The sample size conditions (length * width * height) should generally be less than 10 mm * 10 mm * 5 mm. SEM testing of materials can guide material preparation and component optimization and improve their comprehensive performance. Therefore, in-depth analysis and control of the microscopic characteristics of materials are crucial for material design and optimization. The SEM test results of cement foam filling materials with different water–cement ratios are shown in Figure 7, the SEM test results of cement foam filling materials with different foaming agent contents are shown in Figure 8, and the SEM test results of cement foam filling materials with different gypsum contents are shown in Figure 9.

From Figure 7, Figure 8 and Figure 9, the following can be seen:

(a) The cement foam filling materials prepared with different water–cement ratios can be seen in the electron microscope test, where no matter how much the water–cement ratio is, the main failure is the periodic fluctuation of roughness. When the stress wave acts on the crack tip during the failure process, the stress field at the crack tip periodically changes so that the crack front deviates from the crack’s main plane, leaving wavy patterns on the fracture surface. Cracks do not propagate in a continuously accelerating manner but in a fast–slow–fast–slow alternating manner. The fracture surface of specimens with a smaller water–cement ratio is relatively flat and smooth compared to specimens with a larger water–cement ratio, without obvious plastic deformation marks, and is more prone to brittle fracture. The larger the water–cement ratio, the more tensile textures and extension areas on the fracture surface of the specimen, indicating that the material experienced more plastic deformation during the fracture process, mainly due to the different forms of failure.
(b) The cement foam filling materials prepared with different foaming agent contents can be seen in the electron microscope test that the size and shape of the microparticles of the debris structure on the surface of the specimens prepared with three foaming agent contents are irregular, the particles are arranged in a disorderly manner, the structure between particles is loose, and there are a large number of evenly distributed holes of different sizes. The appearance of microcracks and holes also accompanies the structural plane. As the concentration of foaming agent increases, the size and number of bubbles also increase, which leads to an increase in the internal pores and cracks of the specimen, directly affecting the strength and stability of the material. However, these bubble holes also affect the specimen’s volume expansion and density reduction. Adding a foaming agent to the same mass of dry material can expand the volume several times, but at the cost of losing its strength.
(c) The cement foam filling materials prepared with different gypsum content can see in the electron microscope test that the addition of gypsum promotes the uniform nucleation of pores, which makes the pore structure more closed, low connectivity, and less ellipsoidal pores, indicating that the pore structure is relatively regular, the pore wall is relatively smooth, and the fracture crack propagation is moderate. The selected gypsum dosage is in the low dosage range (0~10%). If the gypsum dosage is too high, it will cause an imbalance in the reaction rate of the foaming agent, and the pores may merge to form irregular bubbles, while microcracks appear on the pore walls. However, when the content is too low (<5%), the micro morphology of gypsum is not much different from that of ordinary cement-based foam filling materials due to too little gypsum content, and some cracks and irregular bubbles appear.

## 4. Characterization of Fly Ash Foam Filling Materials

### 4.1. Mechanical and Transportation Properties Test

**Figure 10:**
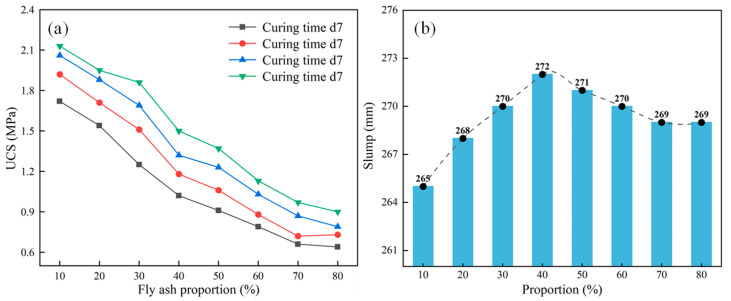
Variations in UCS and slump height with fly ash proportion: (**a**) UCS, (**b**) initial setting time.

The material cost is the main factor affecting the field promotion and application of filling technology. At the same time, the disposal and utilization of solid waste can reduce the damage to the ground environment. Based on the most suitable ratio of the above cement foam materials, this section of the experiment mixes fly ash into the cement foam filling materials at the dosages of 10, 20, 30, 40, 50, 60, 70, and 80%; tests the mechanical and transportation characteristics of the fly ash foam filling materials; and then clarifies the impact of different fly ash contents on the cement foam filling materials and their superior ratio. The material ratio and material characteristics test results of this experiment are presented in Table 8 and Figure 10.

According to Table 9 and Figure 10, the following can be seen:

(a) With the addition of fly ash, when the fly ash content is less than 40%, due to its spherical particles and microaggregate effect, spherical particles can reduce friction between particles, and fine particles can fill the gaps between cement particles, releasing more free water. However, when the fly ash content is greater than 40%, the active SiO_2_ and Al_2_O_3_ in the fly ash react with cement hydration products, which increases the viscosity of the slurry and may slightly reduce its flowability. At the same time, it can also lead to a decrease in the proportion of cement and a weakening of its bonding properties, thereby reducing the material’s mechanical strength. From the perspective of foaming, fly ash can enhance the stability of bubbles, preventing cracking and merging. The synergistic effect of fly ash and foaming agent helps to distribute bubbles evenly. The water demand for fly ash is relatively low, which can increase free water and improve fluidity under the same water–cement ratio.
(b) The fly ash foam filling material’s strength decreases with the increase in fly ash proportion. Still, its strength is higher than that of cement foam filling material with the same proportion. The experimental results show that the UCS gradually decreases with the increase in fly ash content, and fly ash significantly impacts the material’s strength. However, with the gradual increase in fly ash content, this effect gradually weakens, especially when the fly ash content exceeds 60%, and the decrease in strength slows down significantly.

### 4.2. Microstructural Examinations

**Figure 11:**
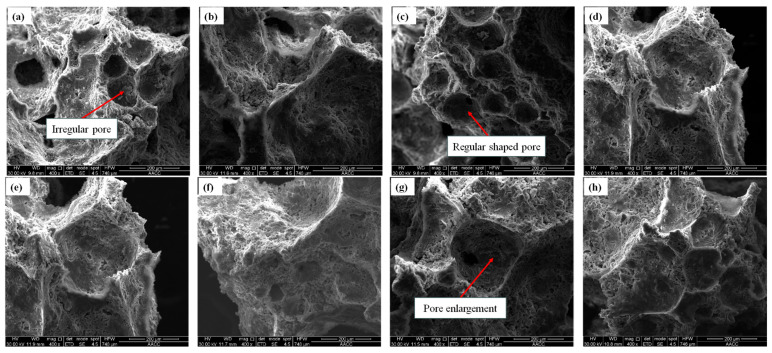
SEM test results of foam filling materials with different fly ash dosages: (**a**) 10%, (**b**) 20%, (**c**) 30%, (**d**) 40%, (**e**) 50%, (**f**) 60%, (**g**) 70%, (**h**) 80%.

Fly ash mixed into cement foam filling materials can promote the continuous hydration reaction of cement, generate more hydration products, improve the micropore structure of materials, and reduce the shrinkage of cement. At the same time, fly ash can also enter the pores. Low-dose fly ash can enhance the material’s impermeability and crack resistance and improve the density of the material. The SEM test results of cement foam filling materials with different fly ash contents are shown in Figure 11.

From Figure 11, the following can be seen:

(a) In the SEM test of foam filling materials prepared with different fly ash contents, it can be seen that no matter how much the content is, the main failure is the periodic fluctuation of roughness. However, when the content is 10% or 20%, the fracture surface is smoother and sharper than that of the high-content specimen. However, when the content of fly ash increases to 30% or more, the fracture surface is more of a periodic fluctuation of roughness, which indicates that the failure mode is more brittle failure. The strength is larger, which is consistent with the law of UCS measured in the test.
(b) The pore shape of the fly ash foam filling material is a regular circle, but the pore distribution is uneven. With the increase in fly ash content, the distribution of pores inside the fly ash foam filling material is more uniform, and the shape is regular. This is because when fly ash is added to the slurry, a better morphological effect is formed between the particles inside the slurry.

## 5. Characterization of Gangue Foam Filling Materials

### 5.1. Mechanical and Transportation Properties Test

**Figure 12:**
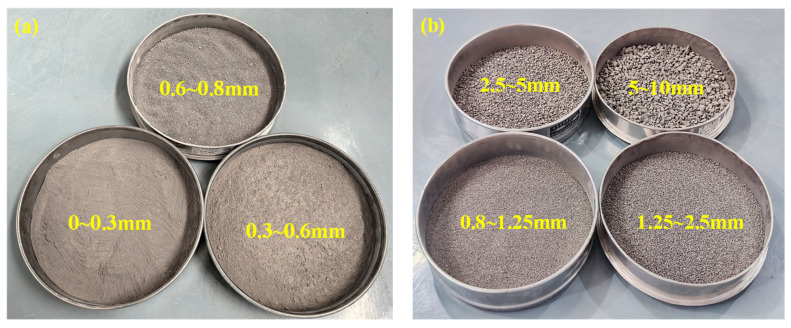
Gangue of different particle size groups: (**a**) small-sized, (**b**) large-sized.

**Figure 13:**
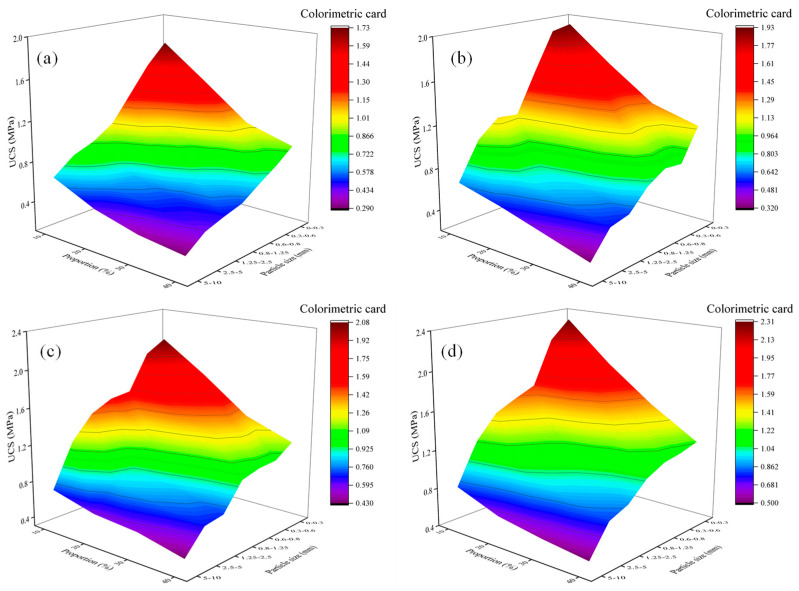
Stress changes in foam materials with different gangue dosages at different curing periods: (**a**) 7 d, (**b**) 14 d, (**c**) 21 d, (**d**) 28 d.

**Figure 14:**
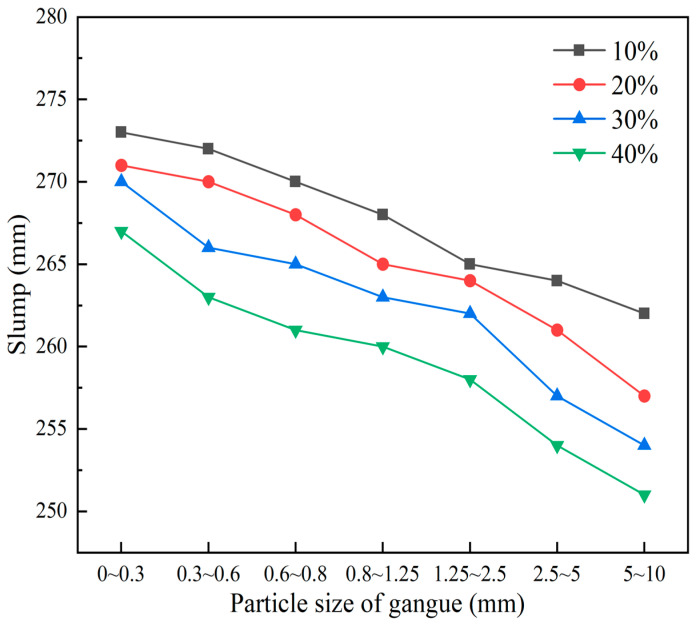
Slum height variation in different particle size groups of gangue cement foams.

Coal gangue is the main solid waste product in coal production, accounting for over 20%. To deal with the solid waste of gangue and reduce the cost of original cement foam filling materials, it is of great significance to study gangue foam filling materials. Since gangue may damage the stable structure of cement-based foam filling materials and lead to material performance degradation, this section selects a 0.45 water–cement ratio to make gangue foam filling materials to ensure that the materials meet the filling requirements. The particle size of gangue in this study is divided into two groups: small particle size: 0~0.3 mm, 0.3~0.6 mm, 0.6~0.8 mm, and large particle size: 0.8~1.25 mm, 1.25~2.5 mm, 2.5~5 mm, 5~10 mm. Different particle sizes of gangue are shown in Figure 12. The mechanical and transport properties of gangue foam filling materials with different gangue particle size additions of 10%, 20%, 30% and 40% were studied. The material ratio and material characteristic test results of this experiment are tabulated in Table 9 and illustrated in Figure 12 and Figure 13. Slum height variation in different particle size groups of gangue cement foams can be seen in Figure 14.

According to Table 9 and Figure 13 and Figure 14, the following can be seen:

(a) With the increase in gangue content and curing period, the moisture in the sample gradually decreases, and its quality and density decrease. With the increase in gangue particle size and content, the slump of foam materials shows a downward trend, and the gangue content has a greater impact on the slump. This is because gangue particles are rougher and have lower fluidity than cement, gypsum, and other materials. From a macro perspective, large particle size gangue should be considered solid particles mixed in the slurry, not fluid. The increase in gangue particle size will amplify the defects in the materials’ flowability, resulting in an increase in gangue particle size and a decrease in slump.
(b) The strength of gangue foam filling material is lower than that of cement foam filling material under the same ratio. This is because the incorporation of gangue makes the originally uniformly mixed cement foam material become a heterogeneous cement, and the contact boundary with foam is prone to producing large bubbles, resulting in large holes after the specimen is dried and molded. Due to the high strength of gangue itself, when disturbed by external forces, it is easy to form a weak structural plane around the gangue particle size, which will form a macro fracture surface if more structural planes are connected.
(c) The higher the content and particle size of gangue, the lower its strength, and it may even have no strength or be unable to demold. However, its UCS increases with the increase in the curing period. At a curing period of 28 days and a content of 10~40%, the UCS of different gangue particle sizes during the process of increasing from small to large are 1.23~2.31 MPa, 1.18~2.12 MPa, 1.15~1.66 MPa, 1.06~1.57 MPa, 0.88~1.47 MPa, 0.8~1.23 MPa, and 0.5~0.81 MPa.

### 5.2. Microstructural Examinations

**Figure 15:**
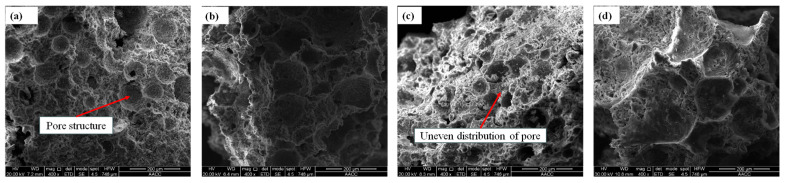
SEM results of foam filling materials with different dosages of gangue of 0~0.3 mm particle size: (**a**) 10%, (**b**) 20%, (**c**) 30%, (**d**) 40%.

**Figure 16:**
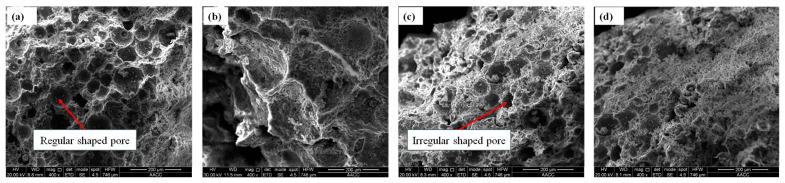
SEM results of foam filling materials with different dosages of gangue of 0.3~0.6 mm particle size: (**a**) 10%, (**b**) 20%, (**c**) 30%, (**d**) 40%.

According to the microstructure electron microscope instrument’s testing principle and the sample particle size test requirements, the samples with smaller gangue particle sizes are selected for testing. Two groups of gangue foam filling materials with gangue particle sizes of 0~0.3 mm and 0.3~0.6 mm were selected for this electron microscope test. The results of the SEM test are shown in Figure 15 and Figure 16, respectively.

From Figure 15 and Figure 16, the following can be seen:

(a) The fracture surface of gangue foam filling material prepared with different gangue content is coarser than that of cement foam filling material in the SEM test. There are many free particles on the surface of gangue foam material, and the strength of gangue particles is large, which will cut the surrounding cement foam material structure, making it easier for the specimen to break from the inside, which is also the reason for the reduction in mechanical properties of the material after adding gangue.
(b) From the perspective of material morphology, the smaller the gangue particle size, the more complete and clear the pore structure under electron microscopy imaging. When the amount of gangue is small, the pore shape is regular and circular, but the pore distribution is not uniform. As the particle size and dosage of gangue increase, the pore shapes inside cement-based foam materials become irregular and diverse, and many pore structures are even damaged to varying degrees. This is because when a large amount of gangue is added to the slurry, the particles inside the slurry are more likely to cause the pores to rupture during mixing and stirring.

## 6. Discussion

**Figure 17:**
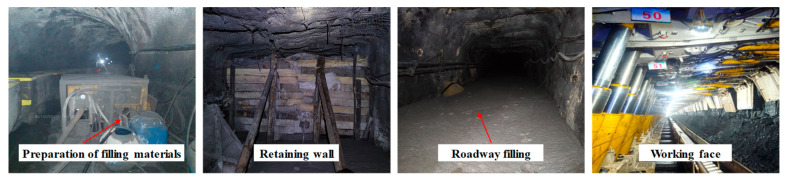
Coal mine roadway filling and on-site application.

Working face passing through an abandoned roadway is a common engineering problem in the underground production process of coal mines. When the geological conditions of the goaf are complex and dense, the impact of mining on the working face can easily lead to large-scale damage to the surrounding rock of the abandoned roadways group, causing a series of safety hazards, such as support roof operation, support bottom leakage, and coal wall lining. Using methods such as working face inclination adjustment and strengthening support for goaf surrounding rock control cannot meet the needs of the multi-layer abandoned roadways group surrounding rock control, and the safe and efficient production of the working face. When the working face inevitably needs to pass through multiple layers of abandoned roadways, various factors such as the influence of mining, advanced support pressure, and the development of the “three zones” of surrounding rock in the goaf will inevitably cause varying degrees of damage and destruction to the abandoned roadways. They can easily lead to safety violations such as roof subsidence, floor bulging, coal wall fragmentation, and surrounding rock damage. The methods of inclined adjustment, undercover or roof lifting in the working face, have complex processes and high requirements for construction accuracy. They are easily affected by factors such as coal seam dip angle and mining pressure manifestation, which can affect the normal operation of equipment in the working face; Traditional roadway support such as wooden stacks and individual structures have limited bearing capacity, especially when encountering complex geological conditions or strong mining pressure, the support effect cannot be guaranteed; Although anchor rods and cables can control the stability of the roof, their control range is limited and they cannot effectively cope with complex geological changes and dynamic pressure on the roof; The filling methods such as high water and paste have some improvement in controlling the stability of the roof, but there are problems such as high material costs. Taking Baodian Coal Mine and Nantun Coal Mine in Shandong Province, China as examples, there are more than 20 abandoned roadways in a single working face. The cross-sectional size, support method, layer, and angle of the abandoned roadways group vary greatly, making it difficult to control the surrounding rock. After using the high-porosity coal mine solid waste materials in this paper, we have achieved safe and efficient production in the working face, which has been applied in more than ten coal mines so far. More than 100 abandoned roadways have a cumulative filling volume of 60,000 m^3^, achieving significant economic and social benefits. Coal mine roadway filling and on-site application can be seen in Figure 17.

## 7. Conclusions

In this paper, the mechanical and transport properties of cement foam filling material, fly ash foam filling material, and gangue foam filling material are studied, and the pore structure and dominant ratio of foam material are analyzed in combination with the material microstructure. The main conclusions are as follows:

(1) The effects of water–cement ratio, foaming agent dilution ratio, foaming agent content, foam stabilizing agent content, and gypsum content on cement foam filling materials’ mechanics, transportation characteristics, and microstructure were studied. The dominant ratio of the comprehensive performance of cement-based foam filling material is determined as follows: the dilution concentration of foaming agent is 20 times, the content of foam stabilizer is 1%, the water–cement ratio is 0.5, the content of foaming agent is 1%, and the content of gypsum is 7%. At this time, the expansion ratio of the material is about three times, the UCS is 1.72~2.25 MPa, the initial setting time is 65~169 min, and the slump is 240~285 mm.
(2) The test results of fly ash foam filling material show that with the increase in fly ash content, the mechanical strength and transportation characteristics of the prepared fly ash foam filling material are reduced. The fracture surface of the foam hole under the electron microscope image is gradually rougher with the increase in the proportion of fly ash. The pore shape of the fly ash foam filling material is a regular circle, but the pore distribution is uneven. With the increase in fly ash content, the pore distribution inside the fly ash foam filling material is more uniform, and the shape is regular. The fly ash content is generally controlled within 30%, which has good mechanical properties. The material expansion ratio is about three times, the UCS is 0.9~2.13 MPa, and the slump is 265~272 mm.
(3) The test results of gangue foam filling material show that gangue foam filling material’s mechanical strength and transportation characteristics decrease with gangue content and particle size. The smaller the particle size of coal gangue, the more complete and clear the pore structure under electron microscopy imaging. When the amount of coal gangue is small, the pore shape is regular and circular, but the pore distribution is uneven. With the increase in coal gangue particle size and dosage, the pore shape inside the cement-based foam material becomes irregular and diversified, and even many pore structures are damaged to varying degrees. When the content of gangue is less than 30% and the particle size of gangue is less than 0.6 mm, it has good mechanical properties. At this time, the expansion ratio of the material is about three times, the UCS is 0.5~2.31 MPa, and the slump is 251~273 mm.

## Figures and Tables

**Table 1:** Chemical composition of fly ash and coal gangue.

Chemical Composition	SiO_2_	Al_2_O_3_	Fe_2_O_3_	CaO	MgO	K_2_O	Na_2_O	Others
Fly ash, wt%	50.6	27.1	7.1	2.8	1.3	0.8	0.2	10.1
Coal gangue, wt%	63.42	17.32	2.62	2.14	1.62	0.6	0.11	12.17

**Table 2:** Gypsum chemical composition.

Chemical Composition	CaO	SiO_2_	Fe_2_O_3_	Al_2_O_3_	MgO	SO_3_	Others
wt%	36.50	6.63	1.69	1.02	2.08	51.08	1.0

**Table 3:** Calcium stearate parameters.

Index	Calcium Content/%	Free Acid/%	Loss on Drying/%	Melting Point/℃	Fineness Through 325 Mesh/%
First-level indicator	6.5 ± 0.5	≤0.5	≤3.0	140 ± 5	≥99

**Table 4:** Experimental results on the cementitious foam density with different stabilizer dosages.

Proportion/%	No	*L*/mm	*W*/mm	*H*/mm	M_0_/g	*ρ*_0_/(g·cm^−3^)
0.6	1-1	71.5	70.8	71.4	232	0.64
	1-2	71.5	70.1	70.0	224	0.64
	1-3	71.4	70.4	70.9	227	0.64
	Average					0.64
0.8	1-1	70.8	70.2	70.4	218	0.62
	1-2	70.4	70.6	70.5	217	0.62
	1-3	70.5	70.0	70.2	217	0.63
	Average					0.62
1	1-1	70.5	69.7	70.4	210	0.61
	1-2	70.2	69.3	70.0	202	0.59
	1-3	70.4	69.9	70.1	209	0.61
	Average					0.60
1.2	1-1	70.9	70.3	70.7	209	0.59
	1-2	70.0	70.2	70.0	203	0.59
	1-3	70.3	70.0	70.6	207	0.60
	Average					0.59
1.4	1-1	71.3	69.4	71.0	205	0.58
	1-2	71.2	71.0	70.5	210	0.59
	1-3	71.0	70.2	70.7	206	0.58
	Average					0.58

**Table 5:** Test results of material properties with different water–cement ratios.

W/C Ratio	Set Time/min	Slump/mm	UCS/MPa				Density/g·cm^−3^
			7 d	14 d	21 d	28 d	
0.40	65	240	1.94	2.06	2.13	2.25	0.73
0.45	73	254	1.75	1.91	2.01	2.06	0.61
0.50	101	265	1.66	1.81	1.92	1.98	0.61
0.55	138	272	1.48	1.54	1.71	1.79	0.58
0.60	169	285	1.37	1.41	1.62	1.72	0.55

**Table 6:** Test results of material properties with different foaming agent dosages.

Foaming Agent Proportion/%	Set Time/min	Slump/mm	UCS /MPa				Density/g·cm^−3^
			7 d	14 d	21 d	28 d	
0.8	97	270	1.77	1.81	1.92	2.08	0.68
1.0	112	266	1.61	1.73	1.87	1.93	0.59
1.2	124	263	1.47	1.60	1.71	1.86	0.55

**Table 7:** Test results of material properties with different gypsum dosages.

Gypsum Proportion/%	Set Time/min	Slump/mm	UCS/MPa				Density/g·cm^−3^
			7 d	14 d	21 d	28 d	
4	101	256	1.77	1.90	2.03	2.20	0.61
5	112	258	1.83	2.05	2.14	2.26	0.61
6	119	259	1.88	2.23	2.39	2.41	0.60
7	125	260	1.97	2.33	2.45	2.52	0.61
8	137	263	1.94	2.20	2.38	2.44	0.61
9	146	265	1.94	2.16	2.32	2.37	0.60

**Table 8:** Test results of the characterization of the fly ash cement foam filling material.

Cement/kg	Fly Ash /kg	Slump/mm	UCS /MPa				Density/g·cm^−3^
			7 d	14 d	21 d	28 d	
9	1	265	1.72	1.92	2.06	2.13	0.62
8	2	268	1.54	1.71	1.88	1.95	0.62
7	3	270	1.25	1.51	1.69	1.86	0.62
6	4	272	1.02	1.18	1.32	1.50	0.61
5	5	271	0.91	1.06	1.23	1.37	0.60
4	6	270	0.79	0.88	1.03	1.13	0.60
3	7	269	0.66	0.72	0.87	0.97	0.60
2	8	269	0.64	0.73	0.79	0.90	0.59

**Table 9:** Test results of the characterization of the gangue cementitious foam filling material.

Gangue Particle Size/mm	Cement/kg	Gangue/kg	Slump/mm	UCS/MPa				Density/g·cm^−3^
				7 d	14 d	21 d	28 d	
0~0.3	9	1	273	1.73	1.93	2.08	2.31	0.66
	8	2	271	1.39	1.59	1.74	1.89	0.65
	7	3	270	1.04	1.28	1.36	1.53	0.64
	6	4	267	0.90	1.15	1.17	1.23	0.63
0.3~0.6	9	1	272	1.53	1.89	1.95	2.12	0.66
	8	2	270	1.26	1.56	1.60	1.78	0.64
	7	3	266	1.01	1.20	1.40	1.45	0.63
	6	4	263	0.77	0.85	1.04	1.18	0.63
0.6~0.8	9	1	270	1.27	1.53	1.56	1.66	0.65
	8	2	268	0.98	1.38	1.47	1.54	0.65
	7	3	265	0.77	1.23	1.30	1.35	0.63
	6	4	261	0.66	0.87	1.03	1.15	0.65
0.8~1.25	9	1	268	1.01	1.15	1.53	1.57	0.64
	8	2	265	0.83	1.04	1.19	1.28	0.63
	7	3	263	0.68	0.97	1.06	1.14	0.64
	6	4	260	0.54	0.77	0.98	1.06	0.63
1.25~2.5	9	1	265	0.88	1.17	1.42	1.47	0.65
	8	2	264	0.63	0.81	0.93	1.18	0.65
	7	3	262	0.53	0.72	0.82	1.01	0.63
	6	4	258	0.49	0.59	0.70	0.88	0.62
2.5~5	9	1	264	0.80	1.01	1.17	1.23	0.62
	8	2	261	0.58	0.78	0.85	1.07	0.64
	7	3	257	0.50	0.66	0.75	0.92	0.63
	6	4	254	0.43	0.55	0.67	0.80	0.63
5~10	9	1	262	0.64	0.66	0.70	0.81	0.61
	8	2	257	0.45	0.56	0.58	0.64	0.59
	7	3	254	0.34	0.44	0.54	0.56	0.60
	6	4	251	0.29	0.32	0.43	0.50	0.59

## Data Availability

The original contributions presented in this study are included in the article. Further inquiries can be directed to the corresponding author.
